# The kin-selected context of dueling in horned aphids: cooperation or conflict?

**DOI:** 10.1093/beheco/araf076

**Published:** 2025-06-29

**Authors:** Keigo Uematsu, Man-Miao Yang, William Foster

**Affiliations:** Department of Biology, Keio University, 4-1-1 Hiyoshi, Kanagawa, 223-8521, Japan; Department of Zoology, University of Cambridge, Downing Street, Cambridge, CB2 3EJ, United Kingdom; Department of Entomology, National Chung Hsing University, 145 Xingda Road, Taichung, 40227, Taiwan; Department of Zoology, University of Cambridge, Downing Street, Cambridge, CB2 3EJ, United Kingdom

**Keywords:** animal contest, cooperation, relatedness, reproductive value, social aphid

## Abstract

We investigated the influence of relatedness on the function of dyadic butting contests over access to a food resource (plant phloem) in the group-living horned aphid *Astegopteryx bambusae* on bamboo leaves. Relatedness between dueling pairs did not differ significantly from that of randomly selected aphid pairs. Microsatellite genotyping showed that the average genetic relatedness between a dueling pair was 0.79 ± 0.12 (mean ± SD, N = 75), with 56% (42/75) of duels occurring between clonal pairs. Butting contests observed in the field lasted longer when the competing aphids were of similar age and when the attacker won, but they involved low costs in terms of time or injury. Neither the duration nor outcome of the contests was associated with the pairwise relatedness, suggesting that there was no kin-discrimination in the butting pair of aphids. 83% (50/60) of the contests between aphids of different ages were won by the older and larger aphid. These results suggest that the aphids discriminate between their opponents on the basis not of relatedness but of size or age. We suggest that the duels in these *Astegopteryx* aphids are not an aggressive fight for resources between different genotypes, but a low-cost method by which the aphids assess each other’s reproductive value, providing an indirect fitness benefit for losing younger individuals that yield a feeding site to older kin. This provides a selective context for the evolution of the young, rather than old, altruistic soldiers that are observed in the open colonies of many cerataphidine species.

## Teaser

Pairs of Astegopteryx aphids fight over food resources - access to plant sap - using frontal horns. We propose that these duels promote cooperation. Field observations and genotyping show that duels occur between genetically closely-related individuals and facilitate the rapid low-cost transfer of food resources from younger, smaller aphids to older, larger aphids. The younger individuals who lose their feeding site gain an indirect fitness benefit by yielding to older kin of higher reproductive value.

## Introduction

Animal combat has always fascinated biologists and the adaptive interpretation of one-to-one (dyadic) fighting has played a pivotal role in clarifying our understanding of the fundamental concepts of evolutionary adaptation: the level at which selection acts and the development of game-theoretic models of animal behavior (Parker 1974; Hardy and Briffa 2013). Dyadic fights have been studied extensively using theoretical models and empirical tests that attempt to determine how the two contestants might assess each other’s combat ability, the relative value of the indivisible resource they are fighting for, and the likely costs of the fight (Kokko 2013; Green et al. 2021).

In group-living animals, however, an additional factor—genetic relatedness—can critically affect the selective landscape of a dyadic fight (Croft et al. 2021; Taborsky et al. 2021; Hardy and Mesterton-Gibbons 2023). If you are fighting a relative, kin selection will mean that your actions will affect not only your fitness but also that of your opponent. For example, reproductive skew in a group, in which dispersal is limited, promotes kin selection for harmful behavior by dominant individuals and helping behavior by subordinates (Johnstone 2008). In addition, when there is significant heterogeneity in age or fecundity, differences in reproductive value might encourage an individual of low reproductive value to help one of higher reproductive value (Uematsu et al. 2013; Hasegawa and Kutsukake 2019; Rodrigues and Gardner 2022). Intense, apparently antagonistic, contest behavior can promote cooperation by enabling the swift transfer of an essential, indivisible resource, thereby increasing the inclusive fitness of the helper. To clarify the function of dyadic contests in a kin-structured group, it is therefore vital to understand the genetic relatedness of the contestants in their natural habitat.

Animals that live in groups and reproduce clonally provide an ideal context in which to spotlight the importance of genetic relatedness on dyadic fighting, since the variation in levels of relatedness is about as high as we can expect to see in nature: ranging from those that are undoubtedly from the same clone to those that are entirely unrelated. Clonality, which occurs in about two thirds of the metazoan phyla (Hughes 1989), gives rise to groups of two main sorts: modular colonies, where the clonal individuals are functionally integrated, as in many colonial benthic invertebrates such as Anthozoa and Bryozoa; and unitary groups, made up of independent clonal individuals, such as aphids and cladocerans (Bell 1982). Fighting behavior has been studied in a number of modular benthic invertebrates, although the fights are often between several individuals in two opposing colonies rather than strictly dyadic fights. However, fighting between independent clonal individuals has been relatively little studied, even though such fights should provide unusually clear situations in which to assess the importance of relatedness to the outcome of dyadic contests.

We report here on a species of aphid, *Astegopteryx bambusae* (formerly *A. bambucifoliae* (Li et al. 2019); tribe Cerataphidini), which provides an exceptionally amenable system in which to look at the kin-selected context of dyadic fighting. Cerataphidine aphids forms a closed gall on the primary host plants (*Styrax* spp.) and winged adults migrate to their secondary host plants, where they form free-living colonies through parthenogenetic reproduction. Several cerataphidine species exhibit dyadic fighting and it is currently unclear why it is only in the Cerataphidini, containing just 115 of more than 5000 species of Aphidoidea (Favret 2025), that fighting for a feeding site has evolved. The answer almost certainly lies in the choice of secondary host-plant (always monocotyledons) in these species and the fact that these aphids tend to feed on relatively mature parts of their host-plants.

**Fig. 1.**
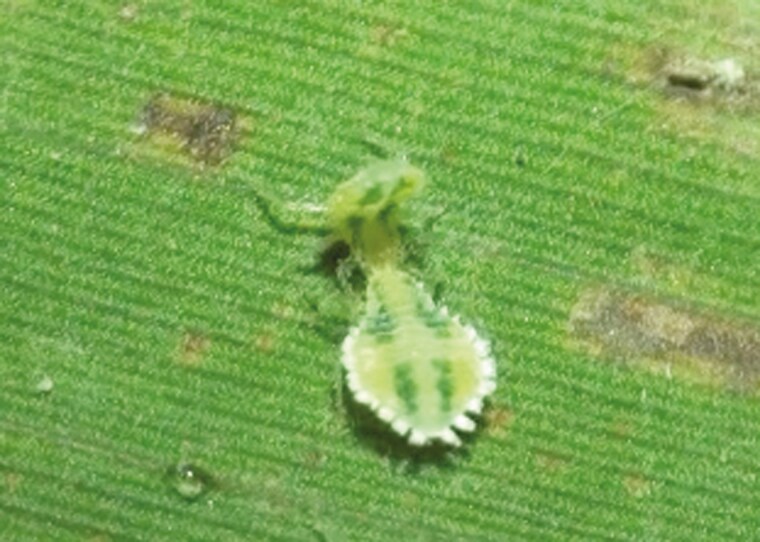
Head-butting in *A. bambusae* aphids. An attacker (front) butts the head of the defender (back) and clasps its body. The defender raises its abdomen and performs headstand. See also Supplementary Video S1.

We know from previous work on this and a closely related species (*A. minuta*) what the aphids are fighting for—access to a feeding site on the leaves of bamboo (Aoki and Kurosu 1985; Foster 1996). This prize is worth the fight. Aphids and plants have been in an arms race over access to phloem for thousands of years and, as a result, it is usually very time-consuming for aphids to find and insert their stylets successfully into the vascular bundle. Morris and Foster (Morris and Foster 2008), however, showed that an aphid (*A. minuta)* that gained access to a ready-made feeding site by winning a fight was able to access the phloem within a matter of minutes. An aphid that wins a particular fight therefore benefits by not having to spend between 20 min and several hours securing a feeding site by other means (Morris and Foster 2008). The fight itself, however, is not costly: fights last for only about 3 min and no damage has ever been observed to be incurred by either of the duelists. In their duel, the attacker is always a free-walking aphid which attacks a feeding aphid, which turns to face the attacker, without withdrawing its stylets during the fight (Fig. 1; Supplementary Video S1).

Previous studies suggest that the butting aphid pairs maximize their direct fitness benefits by assessing their opponent’s resource-holding potential (Aoki and Kurosu 1985; Foster 1996). However, without knowing the pairwise relatedness of the fighting aphids it is not possible to assess the functional significance of what is clearly a common, routine behavior in this and some other aphid species. A previous study, using newly developed microsatellite markers, showed that genetic relatedness within colonies of *A. bambusae* was high (0.54 on average) (Uematsu et al. 2023), indicating that aphids will frequently interact with members of the same clone.

This study aims to answer the following questions.

1. Does an aphid’s size influence (a) whether or not it behaves as an attacker and (b) the size of aphid that it attacks?
2. Does genetic relatedness affect which aphids are targeted by an attacker?
3. Is the duration and outcome of contests influenced by the relative size and/or the genetic relatedness of the contestants?
4. What is the function of the fighting behavior?

## Materials and methods

### Sampling in the field

We defined a “colony” of *A. bambusae* as a group of aphids located on the same leaf of the host plant *Dendrocalamus latiflorus*. We observed and sampled *A. bambusae* colonies on 20, 21 and 29 December 2013 and 23 and 24 April 2014 at Sun Moon Lake, Nantou County, Taiwan. Each colony of *A. bambusae* was observed for a duration of 30 min, focusing on the occurrence of the butting behavior. When an individual aphid (attacker) displayed butting behavior towards another aphid (defender), the age categories of both the attacker and the defender (first instar, second instar, third instar, fourth instar, adult) based on their body size, and the duration of the competition were recorded. We defined the defender’s victory when the attacker ceased butting and left, and the attacker’s victory when the defender withdrew its mouthparts and vacated the feeding site. The time taken for the competition was recorded in minutes. The competing attacker and defender were separately collected by the observer and subsequently preserved in 99% ethanol by an assistant. After 30 min, the entire colony was preserved in 95% ethanol. In total, we observed 23 bamboo colonies (16 colonies in December 2013 and 7 colonies in April 2014) and 111 competitions from 22 colonies, and successfully collected 75 pairs (1 to 7 pairs per colony) from 19 colonies for genotyping. In the laboratory under a dissecting microscope, we confirmed, by measurements of body size, antenna size and horn shape, the age category of the competing aphids which had been recorded in the field simply based on their body size, and counted the total number of aphids in each colony. We also determined the age category of all the aphids in 11 whole colonies. In addition to the 150 competing aphids, 5 to 12 individuals from each of 22 colonies where butting occurred were selected for DNA extraction and genotyping. In total, 370 individuals of *A. bambusae* were genotyped.

### DNA extraction

DNA extraction was carried out using a glass milk extraction method in a microtitre plate as described in Amos et al. (Amos et al. 2016). Each aphid was crushed with a plastic pestle or a pipette tip in 40 μl of lysis solution (10 mM Tris HCl (pH 8.0), 1 mM EDTA, 1% SDS) with proteinase K and incubated at 56 °C for 12 h. The DNA was adsorbed onto flint glass particles in the presence of a 3 × excess of 6 M NaI. Following two ethanol washes, the DNA was eluted in 50 µl of low TE buffer.

### Genotyping

Polymerase Chain Reactions (PCRs) were conducted using Type-it Microsatellite PCR Kit (Qiagen) in 10 µl reactions. PCR conditions were as follows: an initial denaturing step of 5 min at 95 °C, followed by 35 cycles of 95 °C denaturation for 30 s, 60 °C annealing for 90 s, 72 °C extension for 60 s, followed by a final extension step for 30 min at 60 °C. Six microsatellite primers developed in the previous study (Uematsu et al. 2023) were used to generate multilocus genotypes. One primer from each pair was labeled with a fluorescent dye (6-FAM/HEX/NED) as described in Table 1. Following amplification, the PCR product was mixed with the GeneScan 500 LIZ Size Standard (Applied Biosystems) and subjected to fragment analysis. Allele calling was performed using GeneMapper software (Applied Biosystems) and manually checked. Samples with ambiguous genotype were re-run twice and the consensus genotype was taken forward for analysis.

**Table 1.** Polymorphic microsatellite loci with the primer sequences in *A. bambusae*.

Locus	Allele size range (bp)	Primer sequence (5’_3’)	Number of alleles	Polymorphic information content	Fluorescent dye
AbMSAC2	131–153	Fwd-CTAAGGGGAGGGAAATCACG Rev-AAGTGAACCTTCCACGAAAATG	7	0.65	6-FAM
AbMSAC3	177–186	Fwd-GACGATAGCCCTTCTTCGG Rev-ATTTGCAGCTCTCAAGATTTCC	7	0.5	NED
AbMSAC4	89–136	Fwd-ACCACTGCTACGAGATGTTTGT Rev-AAAAGGAGAGCTGACGTGG	12	0.79	HEX
AbMSAC5	171–190	Fwd-CGTAAACGGCCCCAAAAC Rev-AGTCGATGAGATAGCATAAGAACGA	10	0.67	HEX
AbMSAT7	249–259	Fwd-CCAGAGAACTCGTCGTCGTAAT Rev-TCTTCCGTTAAATTCGGTTCAC	4	0.56	HEX
AbMSAT15	134–143	Fwd-TGTCTGTCATCCATTGTCGAA Rev-TGTAGATATGTGGGCCTCTTCA	5	0.58	NED

All primers were developed in Uematsu et al. 2023.

### Data analysis

All statistical analyses were conducted using the R software (R Core Team 2022). To investigate whether or not the age distribution of both the attacking and defending aphids is randomly chosen from the same colony, we generated 10,000 bootstrapped sets from the same colony with replacement. Under the null hypothesis, the observed age distribution should follow the distribution from resampled sets; under the alternative hypothesis (ie age-biased attack or defence), the observed age distribution should deviate significantly from the 95 % confidence intervals estimated from the bootstrapped samples.

To investigate the determinants of butting duration, separate generalized linear mixed models (GLMMs) were employed using the ‘glmer’ command from the lme4 package (Bates et al. 2015). Explanatory variables included age difference, age of attacker, age of defender, and the contest outcome (attacker’s win or defender’s win). The model selection was based on the lowest AIC, with the variance inflation factor (VIF) ensuring no multicollinearity between the explanatory variables (Zuur et al. 2010). Colony identity was set as a random effect. The significance of the fixed effects was determined based on Wald *Z* statistics and *P* values provided by “glmer.”

Different clones were identified using their multilocus genotypes. Pairwise relatedness values among all individuals were calculated following (Queller and Goodnight 1989) using Kingroup (Konovalov et al. 2004) software. The polymorphic information content (PIC, Botstein et al. 1980) of each locus was calculated. To assess the discriminatory power of the set of microsatellite markers for clone detection, the probability of identity (PID) and the probability of identity among siblings (PID_sibs,_Waits et al. 2001) was calculated using R package *poppr* (Kamvar et al. 2014) and *PopGenUtils* (https://github.com/nikostourvas/PopGenUtils). Average relatedness values between competing pairs and randomly selected pairs from the same colony were compared by generating 10,000 bootstrapped sets with replacement, assessing the significance of mean relatedness differences.

## Results

### Observation of butting behavior in the field

We observed and analyzed head-butting in twenty-three colonies of *A. bambusae*. The details of the behavior were as described in previous observations on *Astegopteryx* species (Aoki and Kurosu 1985; Foster 1996). The total number of aphids in the colonies ranged from 82 to 794 (mean ± SE = 377 ± 36). The number of dueling pairs of aphids observed during the 30-min period ranged from 0 to 14, showing no significant correlation with colony size (*t*_21_ = 0.77, *P* = 0.45). The average percentage of aphids in a colony that were observed to be attacking was 3.1 ± 0.6 (mean ± SE, *N* = 75) per hour.

### Relationship between aphid age and fighting behavior

**Fig. 2.**
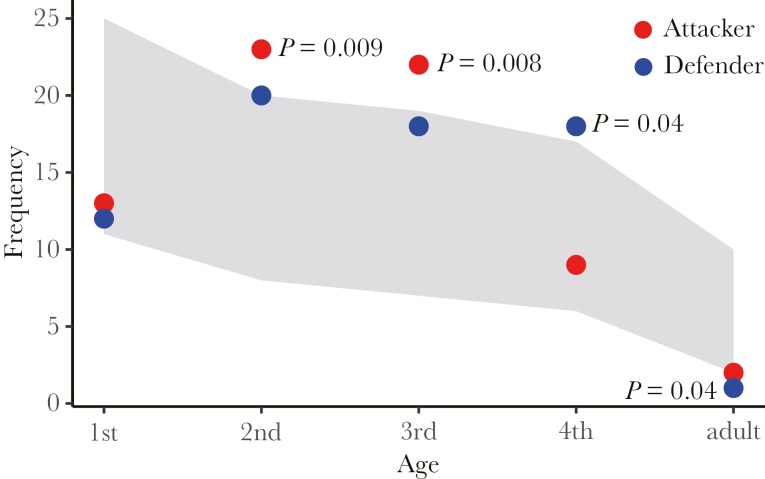
The frequency of butting pairs. A gray band indicates 95% bootstrap confidence intervals from 10,000 bootstrapped sets of the frequency distribution of the five age categories (first to fourth instar and adult) within the observed colonies. Filled circles show the observed frequencies of attackers (red) and defenders (blue). *P* values denote a significant deviation from the 95% confidence intervals.

Of the 111 aphids observed to be attacking other aphids in 23 colonies, only 16 (14%) were fourth instars or adults, the remaining 95 (v86%) were first to third instars (Supplementary Figure 1). This suggests that the younger aphids are more likely than the older ones to initiate contests, but it could simply just be that they are more abundant in the colonies. We therefore measured the numbers of aphids of different ages within 11 colonies and compared this with the observed numbers of aphids that behaved as attackers in these same colonies. The distribution of attacking behavior deviated significantly from random, with the second and third instars being more likely, and the first instars and adults less likely, to behave in attack mode (Fig. 2). Younger instars were more likely to lose when they were defenders (Cochran-Armitage trend test, χ^2^_1_ = 19.2, *P* < 0.0001; Table A1).

### Genetic relatedness between competing aphids

**Fig. 3.**
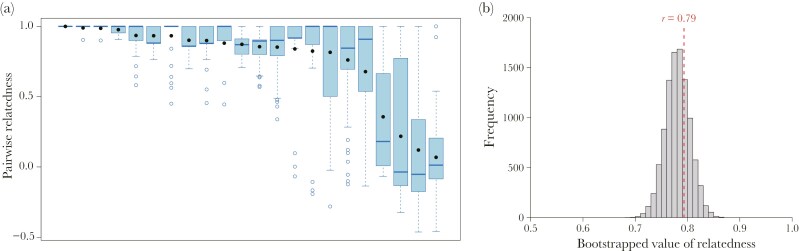
Genetic relatedness between head-butting aphids. (a) Within-colony pairwise genetic relatedness in *A. bambusae* (*N* = 22). Box plots show the range of values, the first and third quartiles and medians. Open circles show outliers and filled circles show means. The box plots are ordered from highest (left) to lowest (right) by their mean value. (b) The distribution of pairwise relatedness within the colonies, derived from 10,000 bootstrapped values, with the red dotted line denoting the observed pairwise relatedness value (mean ± SD = 0.81 ± 0.10) for the 75 butting pairs.

The number of alleles per locus at the six loci used ranged from 4 to 12 (mean = 7.5) and the polymorphism information content of these loci ranged from 0.5 to 0.79 (Table 1). These microsatellite markers have sufficient cumulative power of discrimination among different clones (PID = 9.5 × 10^−6^, PID_sibs_ = 8.5 × 10^−4^,). A total of 107 different clones were recognized based on MLGs from the 370 aphids in the study population. Average pairwise relatedness within a colony ranged from 0.06 to 1.0 (mean ± SD = 0.76 ± 0.08, *N* = 22, Fig. 3a), showing no significant correlation with colony size or butting frequency (vs. colony size: *t*_20_ = 1.68, *P* = 0.11; vs. frequency: *t*_20_ = 0.54, *P* = 0.60). The pairwise relatedness between the competing pairs (mean ± SD = 0.79 ± 0.12, *N* = 75) in the 19 sampled colonies was not significantly different from the distribution of relatedness values between randomly chosen pairs from across the same 19 colonies, weighted according to the number of fighting pairs sampled from each of these colonies (two-tailed bootstrap test, *P* = 0.59; Fig. 3b). Interestingly, 56% (42/75) of the contests were performed by a clonal pair of aphids. Relatedness was not significantly associated with age difference, attacker age, or defender age or outcome of the fight (Table A2).

### Determinants of butting duration and outcome

The attacker won in 57% (63/111) of the contests, with no significant deviation from the expected value of 0.5 (χ^2^_1_ = 2.03, *P* = 0.15). In 60 out of 111 contests where age classes of the pair differed, older aphids had larger body size in all cases, and they were more likely to win (in 83% (50/60) of these contests, the older individual won: χ^2^_1_ = 26.7, *P* < 0.0001).

**Fig. 4.**
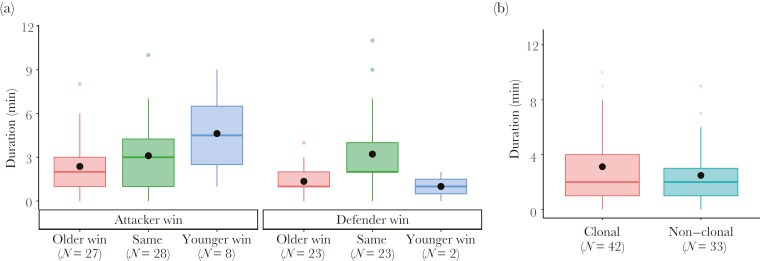
Determinants of the duration of contests. (a) Duration of contests won by attackers (left, *N* = 63) and defenders (right, *N* = 48). (b) Duration of contests between clonal (*N* = 42) and non-clonal (*N* = 33) pairs. Box plots show the range of values, the first and third quartiles and medians. Open circles show outliers and filled circles show means.

The GLMM analysis revealed that age class difference, whether attacker won the contest, and the age of defender were included in the final model (Table 2). Butting duration was significantly longer when age class differences were smaller (β ± SE = −0.26 ± 0.09, *Z* = −2.93, *P* = 0.003), when the attacker won (β ± SE = 0.48 ± 0.15, *Z* = 3.19, *P* = 0.001), and when the defender was older (β ± SE = 0.18 ± 0.08, *Z* = 2.28, *P* = 0.022, Table 2 and Fig. 4a).

**Table 2.** Relationships between butting duration and characteristics of competing aphids.

		β ± SE	95% CI	*Z*	*p*
All (N = 111)					
	(Intercept)	0.63 ± 0.23	[0.18, 1.08]	2.75	0.006
	Age difference	−0.26 ± 0.09	[−0.44, −0.09]	−2.93	0.003^*^
	Attacker age	−0.06 ± 0.08	[−0.22, 0.10]	−0.73	0.467
	Won by attacker	0.48 ± 0.15	[0.18, 0.77]	3.19	0.001^*^
	Defender age	0.18 ± 0.08	[0.03, 0.34]	2.28	0.022^*^
Genotyped (N = 75)					
	(Intercept)	0.41 ± 0.32	[−0.21, 1.03]	1.30	0.194
	Age difference	−0.28 ± 0.11	[−0.51, −0.06]	−2.46	0.014^*^
	Attacker age	−0.01 ± 0.10	[−0.22, 0.19]	−0.12	0.901
	Won by attacker	0.55 ± 0.18	[0.19, 0.90]	3.03	0.002^*^
	Defender age	0.21 ± 0.10	[0.01, 0.41]	2.10	0.036^*^
	Relatedness	−0.04 ± 0.25	[−0.53, 0.45]	−0.16	0.874

^*^Explanatory variable retained in the final model with the lowest AIC.

To investigate the influence of relatedness between the contestants on the duration of contest, we analyzed 75 genotyped pairs engaged in contests. As in the comprehensive analysis that considered all individuals, we included age class difference, the contest outcome (whether the attacker won), and the age of defender in the final model (Table 2). Notably, relatedness was not a significant determinant in the duration of butting (β ± SE = −0.04 ± 0.25, *Z* = −0.16, *P* = 0.87). When categorizing the genotyped pairs into clonal and non-clonal pairs, the duration of butting was not significantly different between these groups (mean ± SD: 3.1 ± 2.6 min for clonal pairs; 2.5 ± 1.9 min for non-clonal pairs; Welch’s t-test, *t*_72.6_ = 1.22, *P* = 0.22, Fig. 4b). The pairwise relatedness was not significantly different between contests won by the attacker (mean ± SD: 0.82 ± 0.35, *N* = 42) and those won by the defender (mean ± SD: 0.76 ± 0.34, *N* = 33; two-tailed bootstrap test, *P* = 0.50). These results suggest that the butting behavior was not influenced by the genetic relatedness between the contestants.

## Discussion

### What are the aphids fighting for?

These field observations provide the most complete account currently available of the dueling behavior of a cerataphidine aphid and our measurements of genetic relatedness allow the adaptive value of this behavior to be evaluated for the first time.

It is clear from previous work on *Astegopteryx* species, and this is probably true for most of the other cerataphidines, that the aphids on the secondary host are fighting for precise feeding sites (Aoki and Kurosu 1985; Foster 1996). Winning a feeding site by fighting can provide significant benefits in terms of the time saved in gaining access to a reliable source of food (Morris and Foster 2008). The costs and benefits of fighting are best evaluated in terms of time: if the attacker wins, it gains the time which would otherwise have to be spent searching for and inserting into a different feeding site, and the defender loses the time that it now needs to spend securing a new feeding site. Conversely, if the attacker loses, it then bears the time cost of finding a new feeding site; the winning defender bears few costs, since even during the fight she could continue to feed. Time not spent feeding really matters to aphids: because of the high nutritional requirements of rapidly developing embryos, their survival rate and fecundity decreases sharply under poor nutritional conditions (Dixon 1987).

### The ecology of fighting behavior

Our observations are consistent with earlier studies on *Astegopteryx* aphids (Aoki and Kurosu 1985; Foster 1996), but are much more detailed and also more reliable since they were entirely carried out under field conditions. Laboratory observations on dueling aphids performed in the previous studies are compromised because the leaves dry out rapidly, which is one of the main reasons why aphids seek new feeding sites, thereby artificially increasing the frequency of fighting (Carlin et al. 1994; Foster 1996). Unusually for a colony of feeding aphids, there is always a small but significant number of aphids (average 3%) moving about, attacking other aphids. The age distribution of both the attacking and defending aphids is highly nonrandom (Fig. 2). Although first instars are commonly seen to be dueling, they in fact attack and defend less frequently than their abundance would predict. These aphids must therefore be able to create—or perhaps find—their own feeding sites, since they are not born with access to the plant phloem (see also (Foster 1996)). Adults are also significantly less likely to be involved in fights than their abundance would predict. This is presumably because other aphids usually choose not to attack them and they are therefore able to retain their initial feeding site. The majority of fights involve second and third instars, which are disproportionately likely to be seen acting as both defenders and attackers.

### The genetic context of fighting behavior

Dyadic competitive interactions are usually studied in the context of the direct fitness outcomes for the contestants. We showed that older and larger aphids tended to win contests and that contest duration was influenced by the age difference between the contestants (Table 2). This finding is consistent with earlier studies on *Astegopteryx* aphids (Aoki and Kurosu 1985; Foster 1996), suggesting that the dueling aphid pairs maximize their direct fitness benefits by assessing their opponent’s resource holding potential.

However, earlier work has shown that these aphids live in colonies consisting of a relatively small number of clones and average within-colony pairwise relatedness was 0.54 (Uematsu et al. 2023). It is therefore essential to consider not just the direct fitness of the contestants, but the inclusive fitness outcomes of the attacker and the defender in any contest. Our genetic analysis established that the attacking aphids, although presented with aphids of a huge range of potential relatedness values, choose to fight with aphids at random: they do not either favor or ignore aphids from the same clone (Fig. 3b). Nevertheless, the average pairwise relatedness of the contesting aphids was 0.79, and 56% of the contests were between aphids from the same clone.

What would be the relative inclusive fitness costs of winning in fights between clone-mates? Fighting itself does not seem to impose any detectable direct costs, such as wounds, on the contestants, and the duration of fighting in this study (Fig. 4) and on *A. minuta* (Foster 1996) (mean: 2.7 min and 3.3 min, respectively) is very small compared to the time required to find and occupy a new feeding site. Therefore, the larger (that is older) attacker should win, since its reproductive value is higher than that of the younger defender: the time penalty of not gaining the feeding site (which could be very high) will impose a relatively higher fitness cost on the attacker, which might be close to reproducing, than on the younger defender. However, if the attacker is smaller, this same argument would imply that the defender should keep hold of its feeding site. This interpretation is consistent with earlier discussions that age or size can indicate future reproduction or reproductive value and can therefore influence altruistic behavior between kin (Cant and Field 2001; Rodrigues and Gardner 2022).

### What are the aphids signaling to each other during a contest?

If this approach is correct, then a duel is not in fact a fight but rather an efficient, low-cost method that the aphids use to estimate each other’s relative reproductive value. Carlin et al. (Carlin et al. 1994) mainly focused on the direct fitness value of fighting in their study, but they did suggest that the butting behavior might also spread information through the colony about the state of desiccation of the host plant. It is empirically very difficult to distinguish between cooperation and aggression in dyadic contexts. The basic difference is that aggressive fights are usually structured around relative resource holding potential, whereas cooperative encounters are about relative reproductive value. In both contexts, but for different reasons, the contests usually include an element of assessment; the older and larger contestant generally wins; and fights last longer when the opponents are more evenly matched in age. In both cases, very young animals rarely attack adults, since they can usually detect, without the need for contact, that these larger individuals are not worth attacking. It seems reasonable to conclude, therefore, that the aphid encounters are cooperative, since the interactions in *Astegopteryx* never involve injury or excessive time-costs. Aphids in this clade are nevertheless perfectly capable of evolving damaging weaponry and longer fights (see for example (Howard et al. 1998; Aoki and Kurosu 2010)).

How might aphids assess each other’s reproductive value? It seems likely that they do this simply by “measuring” each other’s size, which is in general a reliable indicator of age (Foster 1996). Clasping will show the attacker how wide its opponent is and doing a handstand would be a good way for a defender to show long and heavy it is. Head-butting another aphid might also give some idea of its mass.

### Why does relatedness not affect the occurrence, duration or outcome of contests?

Relatedness does not affect which aphids the attackers chose to attack (Fig 3b), or the duration or outcome of a contest (Table 2, Fig 4b). This finding corroborates a previous study on another cerataphidine, *Ceratovacuna japonica* (Carlin et al. 1994), and supports the absence of differences in inter- and intra-species head-butting duration in a mixed colony of two *Astegopteryx* species (Aoki and Kurosu 1985). In fact, there is very little evidence that aphids can discriminate between clone and non-clone mates, in any context, including defensive behavior (Miller III. 1998; Shibao 1999); and mating (Foster and Benton 1992) (but see also Li and Akimoto 2021).

Previous studies of dueling in *Astegopteryx* have assumed that the aphids would benefit from evicting non-clone mates from their feeding sites. But if, as we suggest, these contests are in fact cooperative, then natural selection should favor interactions with clone-mates. It is difficult to envisage how an aphid might be selected to behave differentially towards a clone-mate as opposed to a non-clone-mate, particularly because this differential behavior has never been observed. However, if such discrimination were to evolve, it would likely do so only in contexts where contests with non-clones are considerably more costly—perhaps in terms of injury, lost opportunity, or time—given that the defender gains no indirect fitness benefit by yielding, and the attacker incurs no fitness cost by displacing a non-clonal individual. If clones were identifiable by genetic tags, then an aphid of an alien clone joining a pure clone would almost inevitably engage in costly contests with genetically distinct individuals, either as an attacker or a defender. These interactions would contrast with the lower-cost, more cooperative interactions among clone-mates. This scenario corresponds to a version of Crozier’s Paradox, which posits that when cooperation is mediated by shared genetic tags, the most common tags are selectively favored. Over time, this process can lead to the elimination of rarer tags—including those of invading clones—and ultimately a reduction in the overall diversity of such tags (Crozier 1986; Field et al. 2018). One eriosomatine aphid (*Pemphigus obesinymphae*) is able to detect when they are in a colony of non-clone mates, but only if they have migrated to a distinct aphid colony (Abbot et al. 2001). It would be worth testing whether a *Astegopteryx* aphids that have migrated to totally new colonies behave as though they are surrounded by non-clone mates.

### The evolution of dueling and of the soldier caste in aphids

It has been suggested that the dueling behavior of cerataphidine aphids might have influenced the evolution of the anti-predator fighting behavior of the horned sterile first instar soldiers on the secondary host (Aoki and Kurosu 1985; Aoki 1987; Foster 1996; Stern and Foster 1996). Our observations show that, although the first instars are significantly less likely to attack than predicted from their abundance in a colony, they nevertheless are frequently observed to attack and defeat first-instar defenders (Fig. S1). Our data also provides a more general context for why altruistic behavior might have become focused on the first instars. They have the smallest reproductive value and therefore, if dueling is cooperative, should be more likely to yield their feeding sites to older aphids, promoting larger differences in future reproductive success than those that could be effected by older instars, consistent with theoretical prediction (Johnstone 2008). This could also be the reason why risky dispersal behavior (Aoki 1987) and soldier behavior (Stern and Foster 1996) has become restricted to the first instars in the genera *Ceratovacuna* and *Pseudoregma*.

Duelling behavior has been recorded in all the examined cerataphidine species (Aoki and Kurosu 2010). However, its intensity seems to vary in different species, which could give us clues about how the behavior might have evolved. There appears to be a spectrum of intensity from *Cerataphis brasiliensis*, where dueling is often prolonged (> 14 min), weaponised with long sharp horns, and potentially injurious (Aoki 1987; Howard et al. 1998), through the apparently cooperative behavior described in *Astegopteryx*, to the mild fighting often observed in *Pseudoregma* and *Ceratovacuna* (Aoki and Kurosu 2010). It would be interesting to investigate the intensity of fighting behavior in relation to genetic relatedness in colonies of other cerataphidine aphids in the light of a rigorous phylogenetic analysis.

## Supplementary Material

araf076_suppl_Supplementary_Figure_S1

araf076_suppl_Supplementary_Table_S1

araf076_suppl_Supplementary_Table_S2

araf076_suppl_Supplementary_Video_S1

## Data Availability

Analyses reported in this article can be reproduced using the data provided by Uematsu et al. (2025).
