# Supplementary material for: The kin-selected context of dueling in horned aphids: cooperation or conflict?
Source: Behav Ecol. 2025 Jun 29;36(4):araf076. doi: 10.1093/beheco/araf076 (PMC12264482; doi:10.1093/beheco/araf076)
Supplement: araf076_suppl_Supplementary_Figure_S1 [file araf076_suppl_supplementary_figure_s1.pdf]

Supplementary figure 1

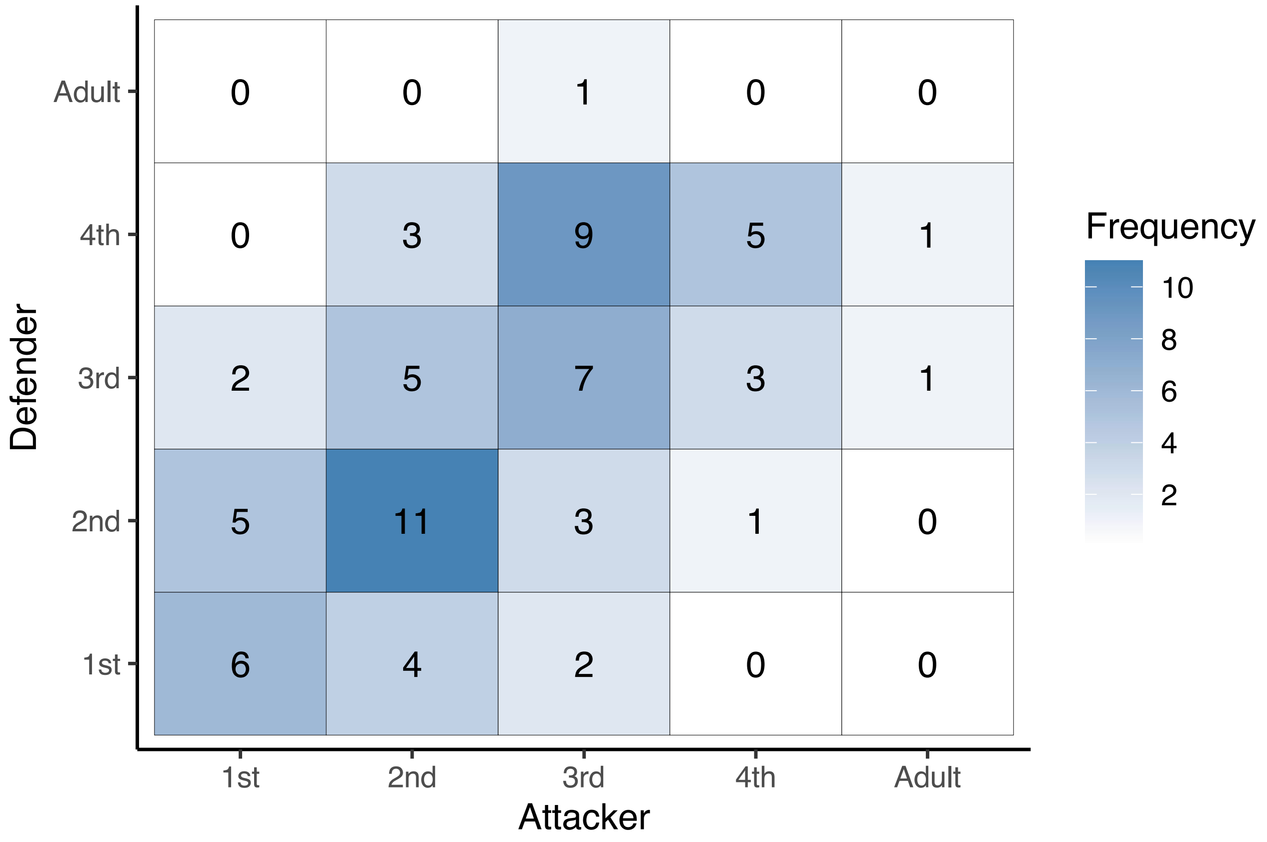

Supplementary figure 1. Pairwise frequency matrix in the age of attackers and defenders. Color density indicate the frequency in the combination of the five age categories (first to fourth instar and adult).
